# Translational Control of COVID-19 and Its Therapeutic Implication

**DOI:** 10.3389/fimmu.2022.857490

**Published:** 2022-03-29

**Authors:** Dejiu Zhang, Lei Zhu, Yin Wang, Peifeng Li, Yanyan Gao

**Affiliations:** ^1^ Institute for Translational Medicine, The Affiliated Hospital of Qingdao University, College of Medicine, Qingdao University, Qingdao, China; ^2^ College of Basic Medical, Qingdao Binhai University, Qingdao, China

**Keywords:** SARS-CoV-2, COVID-19, translation, inhibitor, interferon

## Abstract

The severe acute respiratory syndrome coronavirus 2 (SARS-CoV-2) is the causative agent of COVID-19, which has broken out worldwide for more than two years. However, due to limited treatment, new cases of infection are still rising. Therefore, there is an urgent need to understand the basic molecular biology of SARS-CoV-2 to control this virus. SARS-CoV-2 replication and spread depend on the recruitment of host ribosomes to translate viral messenger RNA (mRNA). To ensure the translation of their own mRNAs, the SARS-CoV-2 has developed multiple strategies to globally inhibit the translation of host mRNAs and block the cellular innate immune response. This review provides a comprehensive picture of recent advancements in our understanding of the molecular basis and complexity of SARS-CoV-2 protein translation. Specifically, we summarize how this viral infection inhibits host mRNA translation to better utilize translation elements for translation of its own mRNA. Finally, we discuss the potential of translational components as targets for therapeutic interventions.

## Introduction

SARS-CoV-2 is a coronavirus with a single-stranded positive-sense RNA genome that can infect a wide range of vertebrates, including wild animals, domestic animals and humans (1–3). Coronaviruses receive their name for the surface of each virion outer membrane is decorated with characteristic “crown-like” spikes that bind to host receptors and confer specificity and infectivity (4, 5). The coronavirus family can be classified into four genera, including alpha-coronavirus, beta-coronavirus, gamma-coronavirus, and delta-coronavirus (6–9). The seven coronaviruses known to infect humans are alpha-coronavirus and beta-coronavirus, while gamma and delta-coronaviruses mainly infect birds. In humans, the seven coronaviruses include four epidemic seasonal coronaviruses (NL63, OC43, 229E and HKU1) and three highly pathogenic human coronaviruses (SARS-CoV, SARS-CoV-2 and Middle East Respiratory Syndrome CoV (MERS-CoV)). Coronavirus disease 2019 (COVID-19) caused by the SARS-CoV-2 virus has become one of the largest and most destructive pandemics in recorded human history. The COVID-19 pandemic has led researchers around the world to use their knowledge to address the problem of infecting humans with SARS-CoV-2, and there are no effective antiviral drugs against this virus (10–16). SARS-CoV-2 belongs to the genus Betacoronavirus of the family Coronaviridae. The genome sequence of SARS-CoV-2 is 80% and 50% similar to that of SARS-CoV and MERS-CoV (17–19). SARS-CoV, MERS-CoV or SARS-CoV-2 infection can develop into a serious, life-threatening respiratory disease and lung damage through infection of bronchial epithelial cells, lung cells and respiratory tract cells (20–22). When SARS-CoV-2 enters into a host cell, it rapidly reproduces using the energy and resources of the host cells, which is critical for deciphering molecular evolution and the controlling the pandemic (23–25).

The infection of SARS-CoV-2 usually results in a large-scale remodeling of gene expression in cells, filling cells with viral transcripts, disrupting innate immune pathways and translating the viral proteins (5, 26, 27). In the multi-level regulatory network, protein synthesis is the focus of control. Since the translation of viral proteins depends on the translation machinery of the cell, coronaviruses have developed a variety of mechanisms to hijack the translation machinery and inhibit the antiviral defense mechanism (28–30). This phenotype is known as host shutoff, which not only increases viral transcription access to ribosomes, but also promotes innate immunity escape. Host shutoff is a key feature of coronavirus infection and has been shown to have a significant inhibitory effect on the innate immune response of a wide variety of pathogenic coronaviruses (including SARS-CoV, MERS, and SARS-CoV-2) (31). This epidemic highlights the need to develop effective antiviral compounds to combat coronavirus infection based on the understanding of the molecular and cellular mechanisms of coronavirus infection (27). In this review, we summarize the strategy used by SARS-CoV-2 to hijack the host translation system to promote viral protein translation.

## Proteins Encoded by Coronavirus Genome

**Figure 1:**
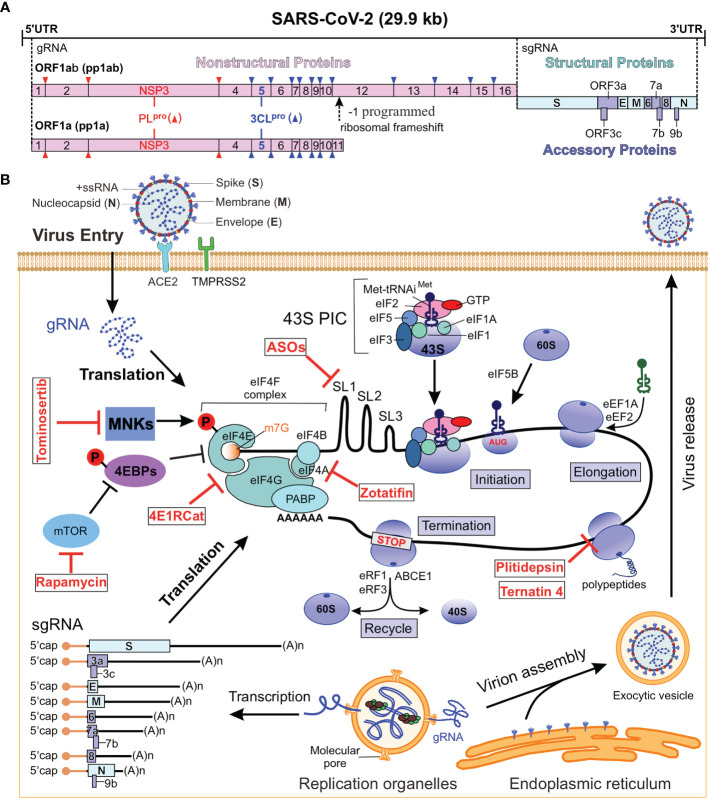
SARS-CoV-2 genome and replication cycle. **(A)** Genome organization of SARS-CoV-2. The single-stranded RNA genome encodes for NSPs, structural proteins and accessory proteins. The sense RNA genome serves as a template for the translation of pp1a and pp1ab, which are cleaved into 16 NSPs. The remaining 30% of the viral genome code at the 3’ end for structural and paraproteins. **(B)** SARS-CoV-2 replication cycle. b SARS-CoV-2 genome and replication cycle. The virus enters the host cell by binding to the ACE2 receptor and releases its genome into the host cytoplasm, whereupon viral proteins are translated by the host ribosome. New virions are assembled and released to complete the life cycle. The red parts are the localization sites of translation inhibitors that can be used for antiviral drugs (**Table 1**). NSPs, nonstructural proteins; ACE2, receptor angiotensin converting enzyme 2; ORF, open reading frame; 43S PIC, 43S pre-initiation complex; MNKs, MAPK-interacting kinases; MAPK, mitogen activated protein kinase; 4EBP, eIF4E binding protein; mTOR, mechanistic target of rapamycin; PABP, poly (A) binding protein; ABCE1, ATP binding cassette subfamily E1; eIFs, eukaryotic initiation factor; eRFs, eukaryotic release factors; eEF1A, eukaryotic elongation factor 1A; Xrn1, 5’-3’ exoribonuclease 1; gRNA, genomic RNA; sgRNA, subgenomic RNAs; ASOs, antisense oligonucleotides.

The SARS-CoV-2 genome is about 30 kb in length that contains 13 open reading frames (ORFs) and encodes 28 major proteins, including 16 nonstructural proteins (NSPs), 4 structural proteins and 6 accessory proteins (**Figure 1A**) (16). In SARS-CoV-2 infected cell, approximately two-thirds of the positive-sense genomic RNA (gRNA) at its 5’ end is directly translated into two polyproteins from the overlapping ORF1a and ORF1b (2, 32). ORF1a is translated into 4,405 amino acids long polyprotein 1a (pp1a), while ORF1b requires a -1 programmed ribosomal frameshift event (-1 PRF) to synthesize pp1b with 7096 amino acids. The pp1a is cleaved into NSP1 to NSP11, whereas the pp1b is sliced into NSP1 to NSP10 and NSP12 to NSP16 (**Figure 1A**) (7, 33–35). The rest of 30% viral genome on the 3’ end is transcribed into 11 subgenomic RNAs (sgRNAs) encoding four structural proteins, including Spike (S), Membrane (M), Envelope (E), and Nucleocapsid (N) proteins, as well as accessory proteins (3a, 3c, 6, 7a, 7b, 8 and 9b) with unknown function (6, 8, 9, 33, 36).

### Nonstructural Proteins

Among the nonstructural proteins, the papain-like protease (PLpro) activity of NSP3, the chymotrypsin-like protease (3CLpro) activity of NSP5, and the RNA-dependent polymerase (RDRP) activity of NSP12 are the core of coronavirus replication (37). Pp1a is translated early and will be cleaved into NSP1 to NSP11 by NSP3 or by NSP5, while pp1ab synthesized through the -1 PRF is cleaved into NSP1-NSP10 and NSP12 to NSP16 (38, 39) (**Figure 1A**). PLpro hydrolyzes the viral polyprotein precursors pp1a and pp1ab at three sites to produce the non-structural proteins NSP1, NSP2, and NSP3 (40, 41). The 3CLpro cleaves viral polyproteins, pp1a and pp1ab, at 11 distinct sites generating NSP4 to NSP16 (40, 41). Inhibitors targeting this enzyme prevent viral replication, making 3CLpro an attractive target for the development of anti-coronavirus drugs. All of these NSPs, except for NSP1 and NSP2, are considered essential for transcription and replication of the viral RNA (42–44). In addition to protease functions, NSPs are involved in modulating the host cell environment, anchoring the viral replication complexes to subcellular domains and driving genome replication, transcription and mRNA processing (2, 38). The SARS-CoV-2 proteins, NSP10, NSP13, NSP14, and NSP16, cap the 5’ end of viral RNA (2). The 5’ cap facilitates viral mRNA stability and translation and prevents detection by host innate antiviral responses. The NSPs form the replication and transcription complex (RTC), which transcribes the viral genomic and sgRNA encoding structural and accessory proteins (45).

### Structural and Accessory Proteins

SARS-CoV-2 generates nine major sgRNAs that encode structural proteins (that is S, M, E and N) and nucleocapsid proteins, as well as other accessory proteins (46) (**Figure 1A**). The structural and accessory proteins are encoded by ORFs located in the downstream of viral genomes. These ORFs are synthesized as a set of 5’-capped subgenomic mRNAs that carry the respective ORFs in their 5’-terminal regions (2). The S glycoprotein (150-200 kDa) is a trimeric transmembrane protein with a predominant ectodomain and a short cytosolic tail. It is cleaved by host proteases into two subunits. The trimeric S-protein on the virus envelope binds specifically to the cell receptor angiotensin converting enzyme 2 (ACE2), which enables the virus to enter susceptible cells, thereby initiating the first step in virus infection (3, 47–49). The M glycoprotein (23-35 kDa) contains a short ectodomain, three transmembrane domains (TMDs), and a C-terminal endodomain (50, 51). The M protein is the most abundant virion protein and plays an essential role during virus assembly (52). The E protein (8-12 kDa) is a minor transmembrane structural protein containing three domains: An N-terminal hydrophilic ecto-domain, a hydrophobic TMD, and then a long hydrophilic c-terminal inner domain. The pentameric bundle of TMD forms iron channels (IC), which probably play a role in the pathogenesis. The N protein (43-50 kDa) has three domains: the N-terminal domain (NTD) and C-terminal domain (CTD) are rich in basic residues that interact with the genome, while domain 3 interacts with the M protein (53). The M protein is a transmembrane glycoprotein that interacts with S, E, and N proteins and is essential for virus morphogenesis and budding (54). Structural proteins are transported through the endoplasmic reticulum to the Golgi secretion pathway, where the viral genome is packaged into budding vesicles, which are then released as new viral particles by exocytosis (2, 17).

Coronavirus accessory proteins are a group of highly variable virus-specific proteins that have limited conservation even within a single species, but their primary role is to help regulate the host’s response to infection and are determinants of viral pathogenicity (12, 14). The accessory genes of ORF3 and ORF8 are the most variation between SARS-CoV-1 and SARS-CoV-2 (7). However, the molecular functions of many accessory proteins remain largely unknown due to a lack of homology with accessory proteins of other coronaviruses or with other known proteins (55). A search for Kozak sequence of individual ORFs for efficient translation shows a required purine A or G at the -4 position in ORF1a, S, M, 7a and 7b, 8 and N and a G at the +4 position in ORF1a, 3a and M (34, 56). An alternative antiviral approach is to target host cell pathways that are essential for viral replication, such as protein synthesis. Among the viral structural proteins and accessory proteins expressed only by a newly synthesized single sgRNA, the S, M, and E proteins are integrated into the viral envelope (membrane) to form viral particles. The interaction between S protein and cell surface ACE2 not only helps the virus to penetrate the host cell, but also activates Death-associated protein kinase 1 (DAPK1), phosphorylates and releases the ribosomal protein L13a (57). These events lead to the blocking of the translation of ORF1a and S mRNA, which depends on the RNA structure of the RNA element (57).

### SARS-CoV-2 mRNA

Viruses can mimic the host’s mRNAs, which contains 5’ cap, 3’ polyadenylated tail, and untranslated regions (UTRs) on the 5’ and 3’ ends to take advantage of the host’s translational machinery (46, 58–60). The 5’ and 3’ UTRs in the genome RNA play essential regulatory roles in virus replication, viral gRNA stability, host immunoregulation, and viral genome encapsulation (61). The SARS-CoV-2 genome has an m7G-cap structure, m7GpppA1, on the genome 5’ end, and a ~30-60-nt-long (47nts in median length) poly(A) tail on the 3’end for viral genome stability and preventing cellular exoribonuclease digestion (16, 62, 63). The 5’ UTR of the SARS-CoV-2 genome is 265-nt long and the 3’ UTR is 337-nt long (excluding the poly(A) tail) (16). The 3’ UTR of SARS-CoV-2 also contains an octanucleotide sequence 5’-GGAAGAGC-3’ with unknown function located at ~70-80 nts of the 3’-end of the viral genome. This sequence is conserved across all genera of the coronaviridae and is a non-essential hyper-variable region (9, 64, 65). However, unlike most mammalian mRNAs, the coronavirus genome has several ORFs between the 5’ end and the 3’ end, both of which contain cis-acting signals involved in RNA replication (11, 12). The transcribed sgRNA contain a common 5’ leader sequence with 72-nt long, which is derived from the 5’ end of viral genome (33, 62, 66) (**Figure 1B**).

### Canonical Protein Translation

During protein synthesis, amino acids are linked into a polypeptide chain, in which specific sequences are translated from the nucleotide sequence of mRNA (67–69). It is generally accepted that the vast majority of coronavirus mRNAs rely on cap-dependent translation to produce proteins (59). A simplified scheme for viral protein synthesis in host cell can be divided into four steps: initiation, elongation, termination, and recycling (**Figure 1B**) (70–72). The initiation step is the key step in regulating the process of protein synthesis. mRNA activation is initiated by the binding of 5’ cap to the eukaryotic initiation factor 4F (eIF4F) complex. As shown in **Figure 1B**, the eIF4F complex consists of cap-binding protein eIF4E, scaffold protein eIF4G and the ATP-dependent RNA helicase eIF4A. During protein translation, eIF4G can interact with poly (A) binding protein 1(PABP1) to circularize mRNA (70).

The 40S ribosomal subunit is the nexus for translation initiation, which recruits activated mRNA through multiple eIFs-mediated process (**Figure 1B**). Under translation initiation conditions, the 40S subunit binds to a variety of eIFs, including eIF3, eIF1, eIF1A, eIF5 and the ternary complex of eIF2-Met-tRNAi-GTP, thereby forming the 43S pre-initiation complex (PIC) (73). The interaction of eIF3 and eIF4G promotes the recruitment of activated mRNA by 43S-PIC to form a 48S initiation complex. The 48S initiation complex scans the mRNA in a 5’ to 3’ direction to find the start codon (usually AUG) and recruits the 60S ribosomal subunit to complete the 80S ribosome translation initiation complex. The second step in cellular protein translation is elongation, which is characterized by the addition of amino acids to the growing polypeptide chain based on the mRNA codon sequence. This process is mediated by the eukaryotic elongation factors (eEFs). Here, eEF1a mediates the cognate aminoacyl-tRNA recruitment to the ribosomal aminoacyl site (A-site), the decoding takes place at the 40S A-site, and the polypeptide chain is transferred from the peptidyl-site (P-site) tRNA to the A-site tRNA. Then eEF2 mediates the translocation of peptidyl-tRNA and deacetylated tRNA from A-site and P-site to P-site and E-site, respectively. The elongation step is repeated until the ribosome encounters one of the three stop codons (UAA, UAG or UGA) that cannot accommodate any of the aminoacyl-tRNA at the A-site. During termination, the eukaryotic release factor 1 (eRF1) and eRF3 recognize a stop codon in the decoding center of the 40S ribosome and release the resulting peptide chain from the peptidyl-tRNA. The 80S ribosome in the post-termination complex is dissociated into 40S and 60S ribosomal subunits by the recycling factor ABCE (ATP binding cassette subfamily E). Taken together, these steps form the basis of ribosome synthesis of eukaryotic proteins (74).

### Non-Canonical Translation

**Figure 2:**
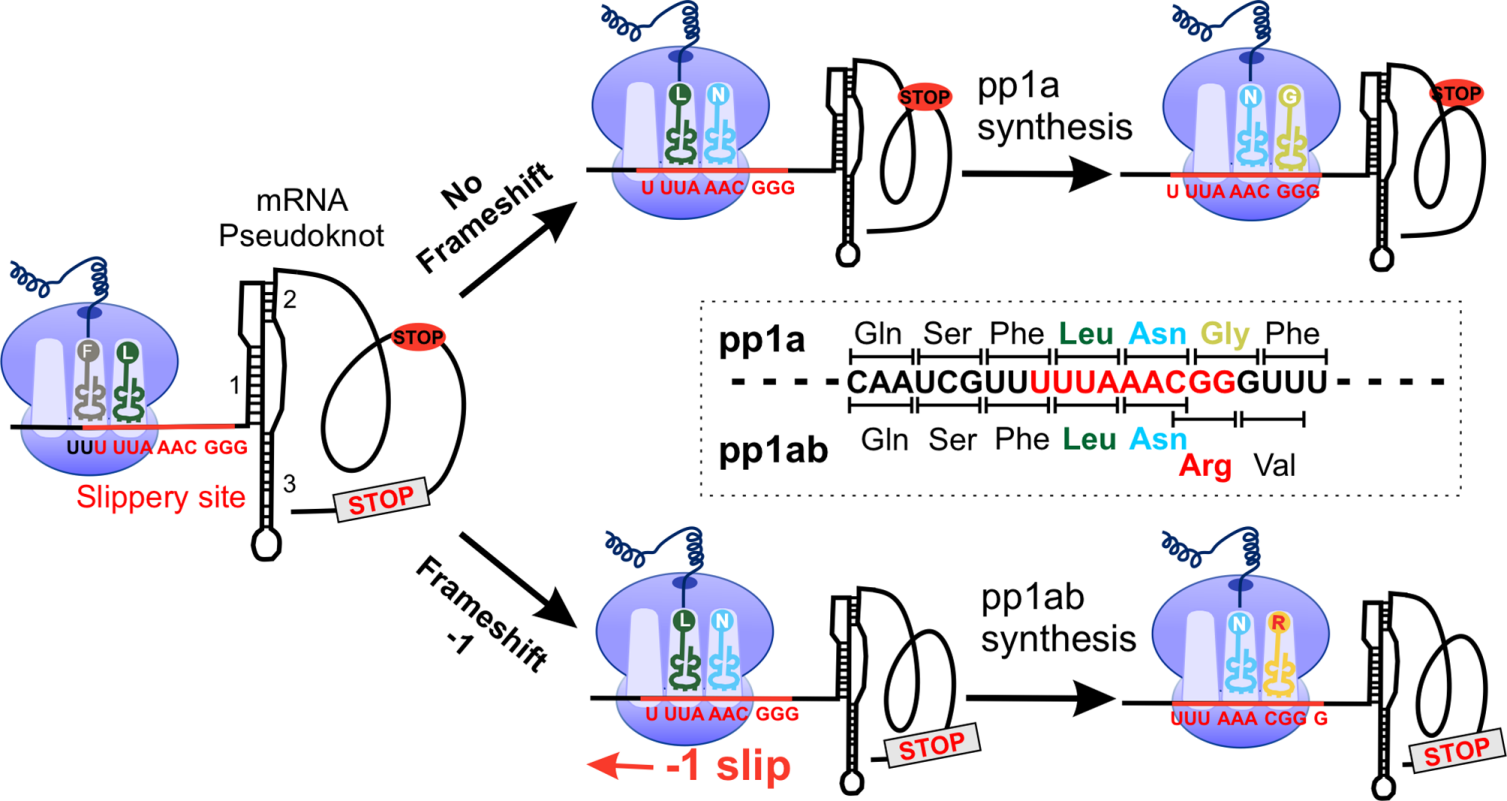
SARS-CoV-2 -1 programmed frameshifting model. The SARS-CoV-2 mRNA pseudoknot interacts with the ribosome, generating tension in the mRNA and pausing translation before the slippery site. After converting “U_UUA_AAC” to “UUU_AAA_C”, translation was resumed at the codon CGG (Arg), resulting in full-length pp1ab. pp1a, polyprotein 1a.

Like many other RNA viruses, coronaviruses use non-canonical translation mechanisms such as -1 PRF and ribosome leaky scanning to expand their coding capabilities and fine-tune the expression levels of certain viral proteins (**Figure 2**) (75, 76). In ribosome leaky scanning, protein translation starts downstream of the annotated start codon, which is driven by the suboptimal nature of the upstream translation initiation signal. The application of this mechanism was found in ORF3c, ORF7b and ORF9b of SARS-CoV and SARS-CoV-2 (75, 77–79). The -1 PRF used in pp1ab translation requires a cis-acting RNA element within the coding region that can redirect the elongating ribosome to shift back 1 base in the 5’ direction back to the first reading frame (80–86). The -1 PRF in the ORF1a/1b overlap region are composed of a slippery sequence “U_UUA_AAC” followed by a “stimulatory” RNA secondary structure, typically a pseudoknot, located 5-7 nucleotides downstream of the slippery sequence (**Figure 2**) (80, 82). The structured RNA pseudoknot that stimulates the -1 PRF at the 3’ end of ORF1a is termed the frameshift stimulation element (FSE) (60, 87). This element interacts with the ribosomal subunit located at the entrance of the mRNA channel and induces translation - 1 pause before FSE. The fully unfolding of this tertiary RNA structure is slow and is thought to promote the displacement of the ribosomal frameshifting on the viral mRNA (83, 85, 88). The -1 frameshifting occurs at this slippery sequence where tRNAs are supposed to dissociate from the mRNA and then shift by one nucleotide in the 5’ direction to a codon into another reading frame “UUU_AAA_C”, thereby generating an alternate gene product by reading through the ORF1a stop codon (81, 84–86).

Compared to the genomic context, the sequence of SARS-CoV-2 FSE is highly conserved to that of SARS-CoV except for a single-nucleotide substitution (C13533A) (8, 11, 89). And the FSE of SARS-CoV and SARS-CoV-2 contains three identical stem architectures, the destruction of which affects the frame shift efficiency (83, 90). This change is located at the loop region in the molecule and is not expected to affect the structure of the three-stemmed pseudoknot (89). This phenomenon indicates that the optimal secondary RNA structure and RNA-RNA interaction in the ribosomal frameshifting signal are important for an effective -1 PRF (81, 86, 91, 92). It is thought that the interaction between specific residues, newly emerged viral polyproteins, and ribosome exit tunnels affects the efficiency of -1 PRF (83, 93). The frameshift efficiency between ORF1a and ORF1b is estimated to be 25% to 75%, which leads to a 1.4- to 2.2-fold overexpression of the protein encoded by ORF1a compared to the protein encoded by ORF1b (33, 94). Studies on SARS-CoV mutants with altered PRF levels have shown that maintaining the expression ratio of ORF1a and ORF1b is essential (95). Repressor of yield of dengue virus (RyDEN) is induced after a SARS-CoV-2 infection, binds to SARS-CoV-2-RNA in infected cells and regulates the efficiency of SARS-CoV-2 -1 PRF (96). In addition, the SARS-CoV-2 FSE is functionally obligate for viral fitness, suggesting that the stimulatory element could be used as a therapeutic target (87, 93, 97). Sun et al. identified merafloxacin, an inhibitor of SARS-CoV-2 -1 PRF, through a high-throughput compound screen and found that it severely prevented other coronaviruses using similar FSEs in cultured cells (98). These results indicate that -1 PRF is a sensitive and effective broad-spectrum antiviral strategy that can be used to combat SARS-CoV-2 and other coronaviruses (98, 99).

## SARS-CoV-2 Suppresses Host Protein Translation

**Figure 3:**
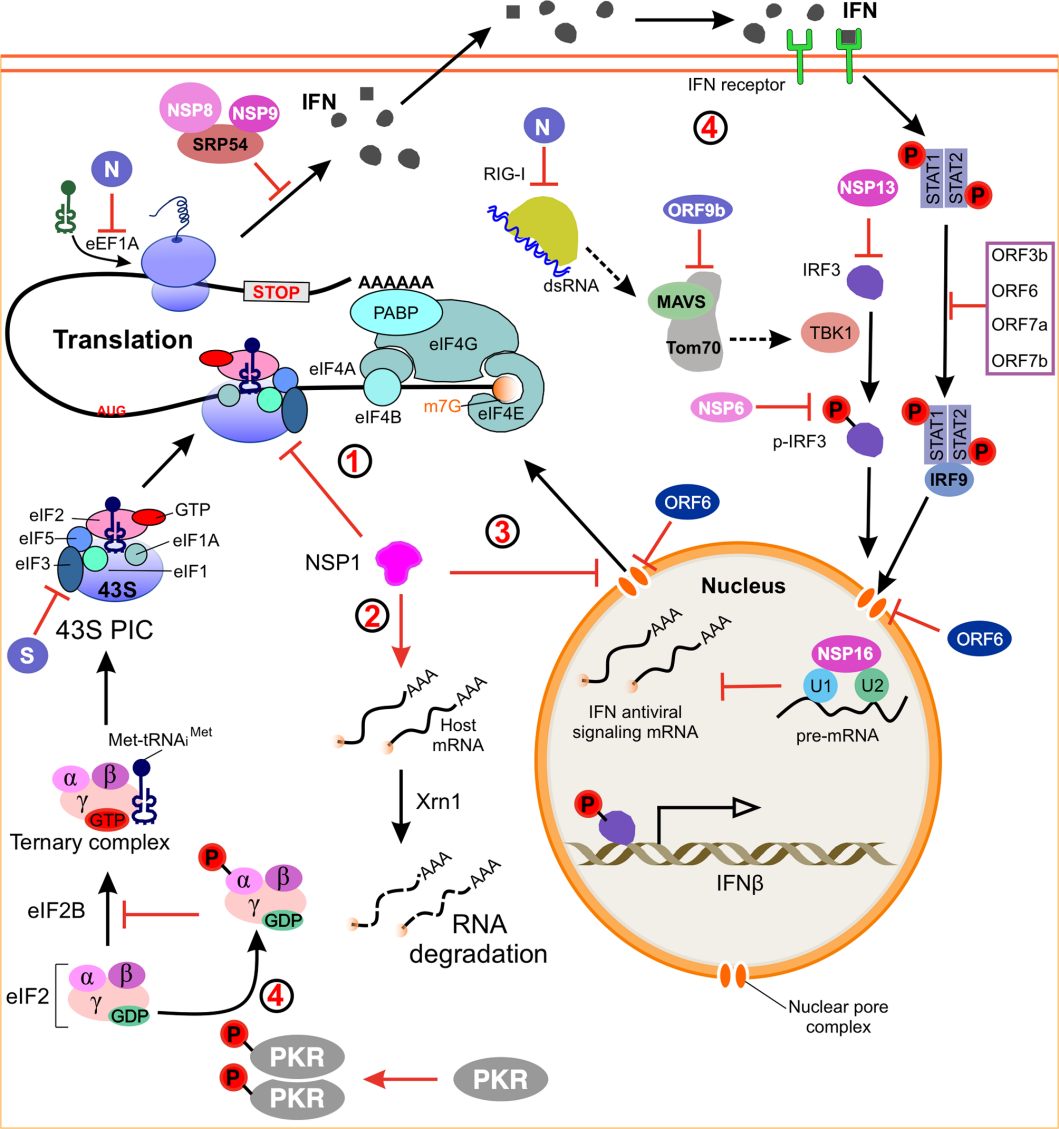
Model of SARS-CoV-2 regulating host gene expression. (1) NSP1 binds to the 40S ribosomal subunit and blocks mRNA entry channels, thereby blocking global mRNA translation. (2) NSP1 induces endonucleolytic cleavage and subsequent degradation of host mRNA *via* the Xrn1-mediated 5’-3’ exonucleolytic mRNA degradation pathway, and these activities are dependent on binding of NSP1 to 40S ribosomes. (3) Inhibition of nuclear mRNA export. NSP1 interacts with the host mRNA export receptor to inhibit nuclear export of cellular mRNA. ORF6 alters the nuclear pore complex by interacting with the export complex thereby preventing the bidirectional translocation of cellular mRNAs. (4) A model how SARS-CoV-2 suppresses host immune responses. NSP1, NSP16, N, ORF6 and ORF9b antagonize the host’s antiviral response and allow the virus replication robustly. SARS-CoV-2 induced activation of PKR inhibits the initiation of eukaryotic translation by phosphorylating the IF2α. dsRNA, double-stranded RNA; IFN, interferon; IRF3, interferon regulatory factor 3; MAVS, mitochondrial antiviral signaling protein; Tom70, outer mitochondrial membrane protein 70; TBK1, TANK-binding kinase 1; STAT1, signal transducer and activator of transcription 1; PKR, protein kinase receptor; eIF2, α subunit of initiation factor 2; SRP54, signal recognition particle 54; RIGs, interferon-stimulated genes.

The complexity of the eukaryotic protein translation is under precise control, but it also enables eukaryotic viruses to exploit or manipulate this process (29). To solve this problem, cells have developed a mechanism to recognize viral infections and then alter the translational ability to limit the production of viral proteins. Viruses, in turn, have developed ways to overcome and even use antiviral defense mechanisms to promote viral protein synthesis. Like many other viruses, coronaviruses are known to globally downregulate host mRNAs translation to allow translation of viral mRNAs (29, 59, 100, 101). SARS-CoV-2 mainly uses four mechanisms to inhibit host mRNA translation (**Figure 3**): (I) the virus NSP1 directly binds to the 40S ribosomal subunit and blocks the mRNA entry channel (30, 38), (II) the infection leads to a host-induced degradation of the cytoplasmic mRNA, which leads to viral transcripts taking over the mRNA pool in infected cells (29), (III) the translation impairment can be caused by inhibiting nuclear mRNA exporting and preventing newly transcribed cellular mRNA from accessing to ribosome (29), (IV) it inhibits cellular translation of cytokines and other factors involved in the innate immune response (29, 102)

### NSP1 Directly Binds the 40S Ribosomal Subunit

Among the 16 NSPs, NSP1 is the first coronavirus protein to be produced in infected cells, containing a structured N-terminal domain (residues 1-125) and a disordered C-terminal tail (residues 126-180) (103). Previous researches on SARS-CoV-1 indicated that NSP1 inhibits host translation by interacting with the 40S ribosomal subunit, inhibiting the formation of 80S (104–106). The NSP1 of SARS-CoV-1 and SARS-CoV-2 has an amino acid sequence homology of 84%, indicating that they could share similar biological properties and functions. Recent structural analyzes showed that SARS-CoV-2 NSP1 binds to the 40S ribosomal subunit and blocks the mRNA entry channel, which leads to the occlusion of mRNA translation *in vitro* and in cells (30, 107, 108). In all instances, the N-terminal globular domain of NSP1 is flexibly positioned on the solvent-exposed surface of the 40S subunit near the entrance of the mRNA channel (107, 109). This domain is anchored by the two C-terminal alpha helices of NSP1. In the free SARS-CoV NSP1 structure resolved by NMR, these two helices are dynamic and unstructured (110). In the NSP1-40S subunit complex, these helices are well resolved and docked in the mRNA entry channel, where they contact the ribosomal proteins uS3 and uS5 and the 18S ribosomal RNA helix 18 (30, 111, 112). The N-terminal and adjacent residues of NSP1 can stabilize the binding of NSP1 and ribosomes and enhance the shutdown function of the host translation (109, 113). As mentioned above, this position on the ribosome is structurally flexible, which adopts open or closed states upon swiveling of the 40S subunit head (73, 114, 115). The presence of NSP1 can also compete with mRNA for binding to the 40S subunit and prevent proper accommodation of mRNA (107). In a recent study, NSP1 was found near the position of the eIF3 translation initiation complex and inhibited protein translation (116). After translation termination, NSP1 can bind to 80S ribosomal complex, suggesting that NSP1 may be involved in translation termination, which has been confirmed by a recent study (117). However, this non-selective translational repression of all host genes can be detrimental to the virus since the viral life cycle invariably depends on host translational factors.

### NSP1-Induced mRNA Degradation

In addition to directly blocking translation, NSP1 induces endonucleolytic cleavage and subsequent degradation of the host mRNA *via* the 5’-3’ exonucleolytically mRNA degradation pathway mediated by Xrn1 (5’-3’ exoribonuclease 1), and these activities are dependent on the binding of NSP1 40S ribosomes (**Figure 3**) (113, 118, 119). Xrn1 is a highly conserved 5’-3’ exoribonuclease that is involved in the degradation of cytoplasmic mRNA (101, 120–122). The NSP1 does not degrade all transcripts equally, nor does it induce the degradation of ribosomal RNA in host cells (101, 104, 123). Weakening of the interaction between NSP1 and ribosome rescues cellular mRNA from degradation and translational repression (113), but the RNA cleavage-deficient mutant still exhibits translational inhibitory activity (13, 106, 124), suggesting that NSP1-induced RNA cleavage can occur following translational inhibition (125). RNA degradation plays an important role in the reconstruction of mRNA pools in infected cells, and these mRNA pools are mainly dominated by SARS-CoV-2 (29). A recent study showed that the N protein directly binds to host mRNAs in the cell, and it is preferable to select 3’ UTR and regulate the stability of the target mRNA (126). Like other viruses, SARS-CoV-2 can hijack the miRNA pathway by producing its own miRNAs, such as CoV2-miR-O7a (SARS-CoV-2 miRNA-like ORF7a-derived small RNA) associates with human Argonaute proteins and represses human targets (127–129).

### Inhibition of Nuclear mRNA Exporting

In fact, compared with mock-infected cells, SARS-CoV-2 infected cells have an accumulation of polyA+ mRNA in the nucleus (130). NSP1 also directly interacts with the host mRNA export receptor heterodimer Nuclear RNA export factor 1 (NXF1)-NTF2-related export protein 1 (NXT1) and is responsible for nuclear export of cellular mRNA (**Figure 3**) (111). The combination of NSP1 and NXF1-NXT1 weakened the translocation of mRNA into the cytoplasm (111), which resulted in a large amount of cellular mRNA being retained in the nucleus during a virus infection. These two distinct functions of NSP1, blocking translation and mRNA export, are performed by different regions at the N-terminal and C-terminal regions of the protein, respectively (30, 112, 131, 132). However, the results of these two examples are conceptually similar, that is the translation of the host mRNA is reduced (111). It is important to note that gammacoronaviruses and deltacoronaviruses do not produce NSP1, due to the lack of NSP1/NSP2 cleavage sites, although similar host attacks are caused by other less obvious mechanisms. In addition, the accessory proteins SARS-CoV and SARS-CoV-2 ORF6 alter the nuclear pore complex by interacting with the export of ribonucleic acid 1 (RAE1) and nucleoporin 98 (NUP98), thereby preventing the bidirectional translocation of cellular RNA (130, 133). NUP98 is a component of the nuclear pore complex, interacts with RAE1, binds to single-stranded RNA and promotes the translocation of mRNA through the nuclear pore complex, but the binding of ORF6 leads to the cytoplasmic localization off RAE1 and NUP98 (134, 135). In cells that overexpress ORF6, the mRNA transporter hnRNPA1, which is thought to chaperone the mRNA through the nuclear pore complex, also accumulates in the cell nucleus (136). Moreover, NSP16 binds to the mRNA recognition domains of U1 and U2 splicing RNAs and inhibits global mRNA splicing when SARS-CoV-2 infection (38).

### Translational Factors as Targets for Host Translation Inhibition

A global analysis of the SARS-CoV-2 protein interaction profile identified 332 protein-protein interactions between the virus and human proteins (10, 137), and another systematic analysis identified 437 proteins with one or more SARS-CoV-2 gene product combinations (138). Some of them are involved in protein translation system (**Figure 3**). N protein binds to eukaryotic translational elongation factor 1A (eEF1A) to induce aggregation of eEF1A, thereby inhibiting the synthesis of the host protein (139). The S protein from SARS-CoV interacts with eIF3f (one of the subunits of eIF3), resulting in suppression of host translation in the later stages of infection (140). NSP8 and NSP9 bind to signal recognition particles (SRP54) and interrupt protein transport during infection, which leads to the degradation of newly translated proteins (38). Similarly, NSP9 also interacts with eIF4H, a factor that enhances the ATP-dependent helicase activity of eIF4A (71, 141, 142). N protein of SARS-CoV-2 was also found to bind the stress granule proteins GTPase-activating protein (SH3 domain)-binding protein 1 (G3BP1) and G3BP2 and another host mRNA-binding protein, including the mTOR-regulated translation repressor LARP1 (La-related protein 1), two casein kinase-2 subunits (CK2) and mRNA decay factors ATP-dependent RNA helicase upstream frameshift 1 (UPF1) and moloney leukemia virus 10 protein (MOV10) (10, 138). LARP1 binds to cellular mRNA containing oligopurine motif (TOP), thereby inhibiting the entry of eIF4E, regulating its stability and translation. On the other hand, since synonymous codons encoding the same amino acid have different concentrations of homologous tRNAs, SARS-CoV-2 codon usage is more relevant to codon usage in human lung, allowing for rapid decoding and protein translation (143–146). In short, all of these strategies ensure that translation of host cell proteins other than viral mRNA is inhibited.

### SARS-CoV-2 Immune Escape

Like other RNA viruses, coronaviruses produce double-stranded RNA through genome replication and mRNA transcription (147–149), which results in the induction of cytokine mRNA transcription, including type I interferon (IFN-I) and IFN-III (31, 150, 151) (**Figure 3**). IFN-I induces the synthesis of interferon-stimulated genes (ISGs) by activating the signal transducer and activator of transcription 1 (STAT1) and STAT2, which are expressed in infected cells and neighboring cells. Meanwhile, SARS-CoV-2 use various strategies to antagonize the host antiviral response allowing the virus to replicate robustly after entering the cell (152–156). The accessory proteins ORF3b, ORF6, ORF7a and ORF7b antagonize the production and signal transmission of IFN-I, while ORF8 disrupts antigen presentation by downregulating major histocompatibility complex class I (MHC-I) (133, 153, 157, 158). In order to block the interferon receptor signal transmission, ORF7a interrupts STAT2 phosphorylation and inhibits the activation of antiviral ISGs (152, 155, 159–161). The C-terminal mutation of ORF7a is usually occur in patient samples all over the world, which leads to significant changes in interferon-stimulated gene expression (161–163). It is hypothesized that there might be redundancy between ORF7a and ORF6 of SARS-CoV-2, which is also believed to inhibit host translation, so that loss of ORF7a *in vivo* can occur at a lower cost of fitness (159, 164). ORF9b expression alone suppresses the innate immune response by interacting with TOM70, a mitochondrial outer membrane protein required to activate the MAVS (mitochondrial antiviral signaling protein) RNA detection linker (158). The MAVS activates TBK1 (TANK-binding kinase 1) and IRF3 (interferon regulatory factor 3) and the subsequent RNA recognition response (158). NSP6 binds TBK1 and inhibits IRF3 transcription factor phosphorylation, whereas NSP13 binds and prevents TBK1 phosphorylation (152, 155). Recent ribosome profiling analysis showed that translation of IFN-I and IFN-III is restricted after SARS-CoV-2 infection (94, 102). The interferon mRNAs are reduced by disrupting translocation of the IRF3 transcription factor into the nucleus, inhibiting their release from the nucleus, and/or triggering their degradation (165, 166). In addition, the NSP14 of SARS-CoV-2 inhibits the protein expression of a large number of ISGs through its global translational inhibitory activity, which offers additional protection against IFN-I response (167–169). NSP3 not only plays a role in inhibiting the host enzyme poly-(ADP-ribose) polymerase (PARP), but also suppresses the expression of interferon genes (170). The IFN-I response is critical for effective protection against viral infections (153, 171), but compared to other respiratory RNA viruses, SARS-CoV-2 is a poor IFN-I response inducer (154, 172).

Another coronavirus protein that affects host translation is SARS coronavirus 7a protein, a multifunctional protein that inhibits host translation, induces apoptosis, and activates p38 mitogen activated protein kinase (MAPK) (173). Activation of p38 MAPK leads to phosphorylation of eIF4E, which promotes translation initiation (174–176). The inhibitors of p38 MAPK, such as ralimetinib, could be considered for testing in humans to combat COVID-19 (176). In contrast, the mechanism by which p38 MAPK inhibits cytokine production and impairs viral replication during SARS-CoV-2 infection is unclear, suggesting that p38 MAPK inhibition may be due to multiple pathogenesis-related mechanisms of COVID-19 (176). However, several recent studies have found that eIF4E is absent from the SARS-CoV-2 RNA interactome, suggesting that translation of SARS-CoV-2 could be eIF4-independent (32, 137, 177, 178). In addition to the IFN-I response, protein kinase receptors (PKR) can trigger translational arrest in infected cells, and PKR is also a type of ISG (179–181). Activation of the PKR inhibits eukaryotic translation initiation through phosphorylation of the α subunit of initiation factor 2 (eIF2α) (151, 182). Before translation initiation, the eIF2αβγ heterotrimer transfers the Met-tRNAi to the 40S ribosomal subunit in the GTP-bound form. After the start of codon recognition, GTP is hydrolyzed, which leads to the release of inactive eIF2-GDP. The guanine exchange factor eIF2B must restore its GTP binding state before another round of translation initiation, which can be inhibited by phosphorylation of eIF2α (183). How SARS-CoV-2 regulates the PKR-eIF2α pathway is still unclear and more research is needed to investigate the possibility of drug therapy related to the IFN response (171). Taken together, NSP1 and other viral proteins inhibit all cellular antiviral defense mechanisms that depend on the expression of host factors, including the interferon response (153, 171, 184). Shutting down these key parts of the innate immune system could promote effective virus replication and immune evasion (13, 105).

### Viral mRNA Escape From Translational Inhibition

In contrast to host mRNA, viral mRNA prevents translation shutdown in the presence of NSP1 (57, 119). It is unclear whether viral mRNA can completely avoid NSP1 inhibition (38, 112). Genes rich in 5’-terminal oligopyrimidines can evade extensive inhibition of its translation by NSP1, indicating that the conserved stem loop in the viral mRNA 5’ leader sequence is necessary for viral gene expression (131, 132, 185). The N-terminal domain of NSP1 binds to the structured 5’ end, as a result of which the C-terminal domain of NSP1 is released from the ribosome mRNA channel (131, 132). However, an earlier study showed that NSP1 inhibits the translation of reporter mRNAs that contain viral 5’ UTRs (112), which means that viral mRNAs cannot easily escape the translational inhibition in the context of 5’ UTR sequences (29, 186). A recent mutation analysis showed that residues in the N-terminal and central regions of NSP1 are not involved in docking 40S mRNA entry channels, but their association with ribosomes and mRNA stabilizes, increasing the restriction on host gene expression and increasing the mRNA contains the SARS-CoV-2 leader sequence, escapes translation inhibition (113). NSP2 interacts with eIF4E2 to employ host translation machinery. In addition, NSP3 can also interact with poly(A) binding protein interacting protein 1 (PAIP1), which can interact with the translation initiation component to enhance translation of viral proteins (187). As a stimulating factor for protein translation, PAIP1 can interact with eIF4A to ensure that only the complete mRNA is selected as the translation template and combined with eIF3 to stimulate translation (188–190). Compared with human RNA, SARS-CoV-2-RNAs have less RNA structure at the 5’ end, so it is more favorable to rapid translation (28, 60).

## Translational Strategies Against SARS-CoV-2

No antiviral drug has yet been shown to be clinically effective in treating COVID-19, and drug development has been hampered by limited understanding of the molecular details of SARS-CoV-2 cell infection. Due to the importance of protein translation in viral replication, translational inhibitors may be an alternative strategy for viral therapy (**Table 1**). As all coronavirus mRNAs rely on cap-dependent translation, the main components of eIF4F cap binding complex-cap binding protein eIF4E, scaffold protein eIF4G and DEAD (Asp-Glu-Ala-Asp) box helicase eIF4A are candidate targets for the treatment of coronavirus (190, 198, 199). Translation initiation factor eIF4H, eIF4A and elongation factor eEF1A1 are essential in viral infections (10, 191, 200), which suggests that translation factors can be used as drug targets for the treatment of SARS-CoV-2 infections and have therapeutic potential (10). Plitidepsin and ternatin4, inhibitors of eukaryotic translation elongation factor 1A (eEF1A), has a potential preclinical effect on SARS-CoV-2 by inhibiting eEF1A, suggesting that translational elongation is critical to viral protein translation (10, 191). Plitidepine has been clinically approved in Australia for the treatment of multiple myeloma (MM), and it can cause toxicity by altering multiple pathways, including arresting the cell cycle, inhibiting cell growth, and inducing apoptosis (191). Previous *in vitro* studies have shown that Emetine inhibits MERS-CoV and SARS-CoV (192, 201). The expected mechanism is to reduce viral RNA and protein synthesis by blocking the interaction between SARS-CoV-2 RNA and eIF4E (192). Zotatifin, a selective eIF4A inhibitor that increases the affinity between eIF4A and specific polypurine sequence motifs, has been reported to inhibit translation of driver oncogenes in lymphoma models (176, 202). This drug has a strong antiviral effect in case of SARS-CoV-2 infection (10, 193). Therefore, inhibition of eEF1A and eIF4A can be extended to other human coronaviruses and beyond unrelated viral pathogens as a strategy for treating viral infections (191). It is predicted that a variety of SARS-CoV-2 proteins will undergo co-translational insertion into the endoplasmic reticulum mediated by SRP and sec61, and SRP19, SRP54, and SRP72 are used as proteins that interact with NSP8 (10, 203, 204). As predicted, several iterative SEC61 inhibitors (including PS3061) have been shown to effectively inhibit the *in vitro* replication of Zika virus and coronavirus (10, 194, 195). Further study needs to be done to evaluate their activities *in vivo*.

**Table 1:** Protein translational inhibitors used for COVID-19.

Name	Structure	Binding sites	Proposed mode of action	References
Plitidepsin	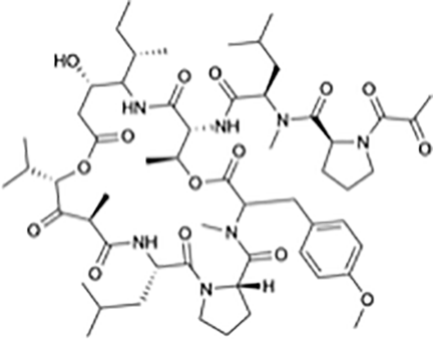	eEF1A	Inhibiting viral protein translational elongation	(10, 191)
Ternatin 4	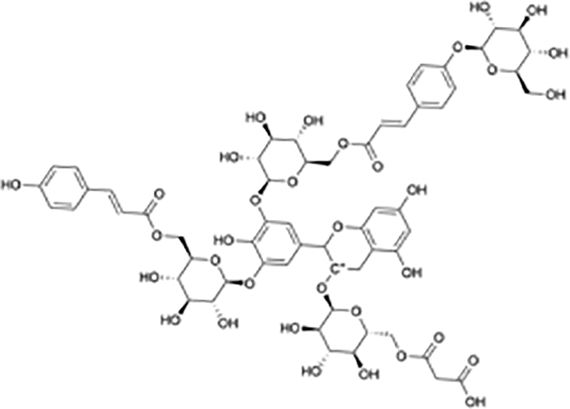	eEF1A	Inhibiting viral protein translational elongation	(10, 191)
Emetine	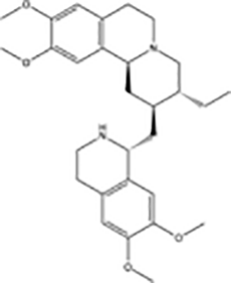	eIF4E	Disrupting the binding of SARS-CoV-2 mRNA with eIF4E	(192)
Zotatifin	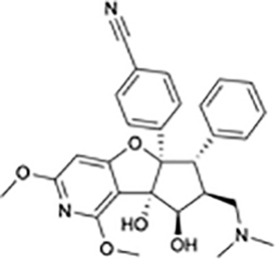	eIF4A	Preventing the virus from unwinding the 5’ UTR	(10, 193)
PS3061	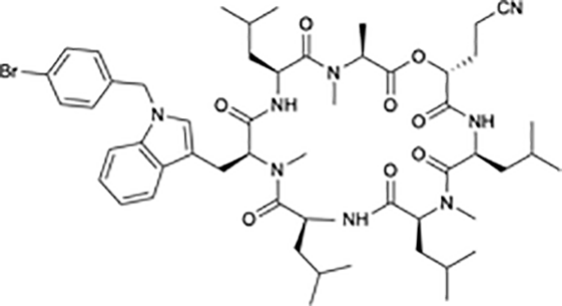	SEC61	Inhibiting the co-translational insertion into the endoplasmic reticulum	(10, 194, 195)
Rapamycin	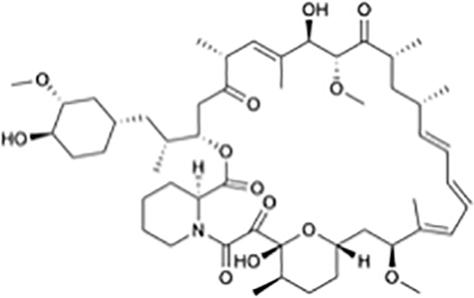	mTORC1	enhances cap-dependent and cap-independent protein translation	(196, 197)
4E1RCat	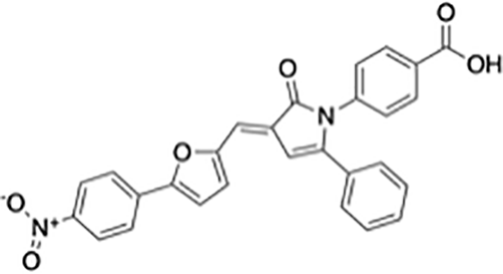	eIF4E:4E-BP1 eIF4E: eIF4G	Preventing the formation of the eIF4F complex and inhibiting cap-dependent viral translation	(10)
Tomivosertib	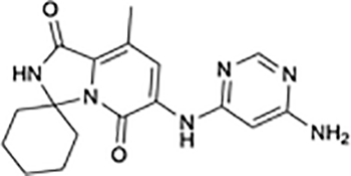	MNKs	Reducing the phosphorylation of eIF4E	(10)

Another small molecule that has been found to regulate host translation is rapamycin, an oral drug that regulates kinases that are involved in host protein synthesis (197, 205, 206). eIF4E binding protein 1 (4E-BP1) and ribosomal protein p70 S6 kinase 1 (p70S6K1) are phosphorylated by mechanistic target of rapamycin complex 1 (mTORC1), which enhances cap-dependent protein translation (196, 197). 4E1RCat is a dual inhibitor of the interaction of eIF4E: 4E-BP1 and eIF4E: eIF4G, which prevents the formation of the eIF4F complex and inhibits cap-dependent viral translation (10). The study of White et al. suggests that translation inhibitors may have promise in treating patients with mild or moderate COVID-19 (191). Tomivosertib, an inhibitor of MNKs (MAPK-interacting kinases) reducing the phosphorylation of eIF4E, has recently been highlighted for the treatment of SARS-CoV-2 infection (10). Host targeting has discernable advantages, including creating a higher threshold for virus resistance and providing broader protection for different strains of the virus (10, 195). It was previously shown that coronaviruses are very sensitive to translation inhibitors, although these inhibitors may also affect host translation (191, 207, 208).

Targeting conserved RNA structures and sequences of SARS-CoV-2 is an alternative approach to inhibiting viral infection and progression (209, 210). The most well-known examples are antisense oligonucleotides (ASOs), which contain modifications at their positions, such as 2-O-methyl (2-OME), 2-O-methoxy (2-MOE), locked nucleic acid (LNA), morpholino, or other nucleotide modifications, which may increase RNA base pairing, metabolic stability, and/or delivery (209, 211, 212). Circular RNAs (circRNAs) can also be engineered as antisense RNAs to disrupt SARS-CoV-2 genome expression and viral proliferation (213). These antisense-RNAs form stable hybrids with their target RNAs, which cause target RNAs cleavage/degradation or block mRNA processing or translation (213–215). Antisense DNA oligonucleotide forms a hybrid with the target RNA and induces cleavage of RNA by RNase H, an endonuclease that cleaves the RNA-DNA strands, limiting the synthesis of the encoded protein (212). Another study showed that in a pseudovirus infection model, 2’-OME/SP-ASO conjugated with four 2’-5’-oligonucleotides that can induce RNase L-mediated cleavage and degradation of SARS-CoV-2 envelope and spike, thereby effectively inhibiting the spread of the virus (216). The stem-loop 1 (SL1), a highly conserved sequence in the SARS-CoV-2 5’ UTR, is necessary and sufficient to bypass NSP1-mediated shutdown, leading to the design of LNA ASOs targeting this sequence and enabling translational shutdown of virally susceptible NSP1, thereby effectively inhibiting viral replication (214, 217). The combination of cryo-electron microscopy and molecular modeling reveals the tertiary structure of the SARS-CoV-2 frame-shift stimulus element (87). RNA-modified ASOs that target the structure of this element can disrupt translational frameshifts and thereby inhibit viral replication (87, 210).

## Discussion and Prospect

Currently, there are lots of information suggesting that coronaviruses have evolved a number of mechanisms to control viral and host gene expression at the post-transcriptional level. This review not only provides an overview of the experimental research in driving and controlling mRNA translation of virus and host cells in coronavirus infected cells but also summarizes the new targets on translation system for therapeutic intervention. Translation is closely related to other cellular processes (such as RNA degradation), which raises the question of how the global environment affects or will affect these viral mechanisms. To answer this question, contributions from many fields are required, including virology, structural biology, biochemistry, and cell biology. Excitingly, new tools are emerging that can help solve these problems. For example, advances in structural methods such as cryo-electron microscopy will enable the visualization of large complexes including viral RNA and translational machine, which is useful for studying translational status during viral infection. In particular, recent advances in single-cell protein genomics and single-cell signal visualization may provide important information about the contribution of SARS-CoV-2 infection to the regulation of their own replication.

## Author Contributions

DZ, LZ, YW, PL, and YG conceived and discussed the outline of the review. DZ and LZ wrote the first draft of the manuscript. YG, YW, and PL edited and finalized the manuscript. DZ generated all figures and table. All authors contributed to the article and approved the submitted version.

## Funding

DZ was supported by National Natural Science Foundation of China (Grant: 3187040505).

## Conflict of Interest

The authors declare that the research was conducted in the absence of any commercial or financial relationships that could be construed as a potential conflict of interest.

## Publisher’s Note

All claims expressed in this article are solely those of the authors and do not necessarily represent those of their affiliated organizations, or those of the publisher, the editors and the reviewers. Any product that may be evaluated in this article, or claim that may be made by its manufacturer, is not guaranteed or endorsed by the publisher.
